# Impact of Medical Therapies on Cardiovascular Risk Factors in Acromegaly

**DOI:** 10.7759/cureus.86106

**Published:** 2025-06-16

**Authors:** Robert Wekesa, Sriradha Chatterjee, Omar Alhiraki, Aisha Sheikh

**Affiliations:** 1 Acute Medicine, Lincoln County Hospital, Lincoln, GBR; 2 Endocrinology, University of South Wales, Cardiff, GBR

**Keywords:** acromegaly, cardiovascular risk, medical treatments, pegvisomant, somatostatin analogs

## Abstract

Acromegaly results from excess growth hormone production and has a significant burden of cardiovascular comorbidities. Various medical therapies have been shown to be efficacious in achieving disease control along with surgery and radiotherapy. However, the impact of these medical therapies on cardiovascular risk factors remains unclear. The overall objective of this review was to assess the impact of various medical therapies used for acromegaly on blood pressure, glucose metabolism, left ventricular mass, and lipid profile.

A systematic narrative synthesis was conducted in accordance with PRISMA recommendations (PROSPERO registration number CRD42024609974). A comprehensive search of electronic databases was conducted in MEDLINE, OVID, ProQuest Central, Scopus, and Web of Science. The search covered studies published in English over the last 20 years on acromegaly medical treatments and cardiovascular risk factors. The review included randomized control trials, non-randomized control trials, case-control studies, and cohort studies. Only original before-and-after treatment studies assessing systolic and diastolic blood pressure, fasting plasma glucose, HbA1c, left ventricular mass, and lipid profiles were selected. Reviews, case reports, uncontrolled trials, and studies addressing other cardiometabolic parameters were excluded. To evaluate the methodological quality of the included studies, the risk of bias was assessed using the standardized critical appraisal tools developed by the Joanna Briggs Institute (JBI).

A total of 4777 articles were retrieved, out of which 20 met the inclusion criteria for the qualitative analysis after data extraction. The growth hormone receptor blocker pegvisomant was associated with a significant post-treatment reduction in both fasting plasma glucose and HbA1c. The long-acting somatostatin receptor ligand pasireotide, on the other hand, was associated with an increase in these glucose parameters. All the medical therapies were associated with a decrease in left ventricular mass when disease control was achieved over a sufficient period. A significant reduction in lipid parameters was also observed in patients treated with somatostatin receptor ligands. However, the impact of these agents on blood pressure control was confounded by concurrent antihypertensive therapies.

## Introduction and background

Acromegaly is characterized by excess growth hormone (GH) and insulin-like growth factor-1 (IGF-1) production. The excess GH arises from a pituitary adenoma in more than 90% of cases. Other rare causes of excess GH activity include ectopic production, excess growth hormone-releasing hormone (GHRH), and GH receptor mutations. According to Chanson & Salenve [1], acromegaly has an estimated prevalence of 40-70 cases per million population, an incidence rate of 3 to 4 cases per million, and affects both males and females equally. In accordance with the treatment guidelines by Ogedegbe J et al. [2], the diagnosis of acromegaly is established by elevated age-specific IGF-1 levels and a failure to suppress GH production after an oral glucose tolerance test (OGTT). The excess GH and IGF-1 production then result in several cardio-metabolic abnormalities that cause significant morbidity and mortality in these patients (Puglisi S et al.) [3].

According to the review of cardiometabolic risks in acromegaly by Puglisi, S, et al. [3], the prevalence of hypertension has been estimated at 11-54%, diabetes at 16-56%, and sleep apnea syndrome at 45-80%. Lipid abnormalities were estimated at between 33% and 47%. The study by Vitale G, et al. [4] estimated the prevalence of left ventricular hypertrophy at 50 to 80%, with a higher prevalence in patients with a long duration of disease.

As per the treatment guidelines by Ogedegbe J et al. [2], the recommended first-line treatment for acromegaly is surgery. However, patients who fail to achieve disease control or those unsuitable for surgery have the option of radiotherapy or medical therapy. The available medical therapies are dopamine agonists (cabergoline), first-generation somatostatin receptor ligands (SRLs), also referred to as SSAs (octreotide and lanreotide), the second-generation SRL (pasireotide), and GH-receptor antagonists (pegvisomant) used singly or in combination (Ogedegbe J et al.) [2].

Cabergoline is a selective dopamine-2 (D2) receptor agonist, commonly used to treat hyperprolactinemia, which has also found use in acromegaly (Ershadina N, and Tritos N) [5]. It has a reported efficacy of up to 34%, and its side effect profile includes headache, nasal congestion, and orthostasis (Sandret L et al. [6]).

Somatostatin receptor ligands (SRLs) activate somatostatin receptors (SSTRs) expressed in the pituitary as well as other parts of the endocrine system. First-generation SRLs octreotide and lanreotide predominantly activate SSTR-2, SSTR-3, and SSTR-5 and have reported efficacy of up to 56% GH and IGF-1 control (Carmichael, et al.) [7]. Clinical response to the first-generation SRLs may be predicted by certain germline abnormalities, somatostatin receptor expression by the adenoma, tumour histopathological features, and certain radiological features, Gadelha et al. [8]. A significant proportion of patients treated with first-generation SRLs also achieve tumour shrinkage, making them a viable choice for pre-operative treatment (Caron P et al. [9]).

The second-generation SRL pasireotide activates a broader spectrum of somatostatin receptors (SSTR-1, SSTR-2, SSTR-3, and SSTR-5) and has a demonstrated superiority in disease control over the first-generation SRLs. Bromstein M et al. [10]. The growth hormone receptor (GHR) antagonist Pegvisomant is currently the most efficacious, achieving IGF-1 control in up to 60% of patients sub-optimally controlled by SRLs. Trainer P et al. [11]. Due to the suppression of the negative feedback loop of IGF-1 on the pituitary gland, pegvisomant has a modest risk of increasing tumour size. Other side effects include an increase in liver enzymes and lipodystrophy at the injection site (Van der Lely et al.) [12]. According to the 13th acromegaly consensus statement by Giustina A et al. [13], the choice of a single or combination of medical therapies depends on the disease characteristics (tumour size and GH/IGF-1 levels), costs, preferred route of administration, and pre-existing comorbidities such as diabetes.

Disease control by reduction of IGF-1 levels has been shown to result in a decrease in acromegaly-related mortality to that experienced by the general population. Holdaway M et al. [14]. This decreased mortality has been putatively hypothesized to be the result of improvement in the burden of cardiometabolic profiles. Patients who achieve remission through surgery alone, for example, have been shown to have an improvement in glycemic parameters, systolic blood pressure, and better lipid profiles (Reyes-Vidal C et al. [15]). 

Previous reviews have reported varying impacts of the medical therapies on cardiometabolic profiles in acromegaly patients, which have been attributed to the different pharmacodynamic properties of the agents. This review therefore sought to consolidate current evidence for improvement or deterioration of cardiometabolic abnormalities in acromegaly patients undergoing medical therapy. The key parameters assessed are the impact of medical therapies on glycemic indices, blood pressure, left ventricular mass, and lipid profile.

## Review

Methods

This systematic review was performed according to Preferred Reporting Items for Systematic Reviews and Meta-Analysis (PRISMA). The study protocol was published in the PROSPERO database under the registration number CRD42024609974. The study was conducted in accordance with the ethical considerations of the Helsinki Declaration. The protocol ethics approval was obtained from the USW Ethics Review Board before the commencement of the study.

Search Strategy

A comprehensive search was done of all medical research databases (Medline, Embase, Scopus, and Web of Science) for studies that have evaluated the impact of medical acromegaly therapies on cardiometabolic risk factors. A direct reference search from review articles was also done to identify similar studies. The population of interest comprised all acromegaly patients, the intervention was all medical treatments for acromegaly, the comparator was before and after treatment assessment against a control group, and the outcome of interest was a change in the listed cardiometabolic parameters.

Keywords for the search strategy were ‘acromegaly,’ cardiovascular risk*’, and ‘acromegaly treatments.’ 

Inclusion and Exclusion Criteria

Original studies published in the English language over the last 20 years that assessed cardiometabolic risk factors in acromegaly patients on medical therapy were included. With regard to study design, we included randomized and non-randomized controlled trials and cohort and case-control studies only. The review included studies that addressed the following cardiovascular outcomes: HbA1c, fasting plasma glucose (FPG), glucose tolerance (GT), systolic blood pressure (SBP), diastolic blood pressure (DBP), and left ventricular mass index (LVMI). Lipid profile parameters of interest were total cholesterol (TC), triglycerides (TG), low-density lipoprotein cholesterol (LDLc), and high-density lipoprotein cholesterol (HDLc).

The review excluded case reviews, case reports, opinion pieces, and trials for patients achieving control by surgery alone. Further, studies that assessed secondary outcomes such as insulin resistance, beta-cell function, left ventricular function (LVF), cardiac rhythm abnormalities, arterial stiffness, lipid metabolites, adiposity, or inflammatory markers were also excluded. Others excluded were animal studies, studies that were published more than 20 years ago, and those that were unpublished.

Study Selection and Data Extraction

All identified study results were exported to the ENDNOTE citation manager and shared with the two study selectors. A four-stage screening was done in the following order: Stage 1, removal of duplicate articles by ENDNOTE citation manager. Stage 2 involved title screening to identify irrelevant articles. Stage 3 was a full abstract review, while Stage 4 involved a full article review, including a quality of evidence assessment.

Study selection was done by two independent selectors based on the inclusion and exclusion criteria. A consensus meeting was held to resolve inconsistencies between the two selectors. Data extraction was done by the principal investigator. The following data was extracted from the individual studies: author(s), study design, year of study, number of participants, participant characteristics, type of medical therapy (single/combination), cardiometabolic indices before and after treatment, effect size, and confidence intervals.

Quality of Evidence Assessment

The quality of evidence was assessed using the JBI critical appraisal tools for RCT, non-RCT, cohort studies, and case-control studies (Maola S et al.) [16]. A total JBI score of 7 and above was considered an acceptable risk of bias.

Results

**Figure 1:**
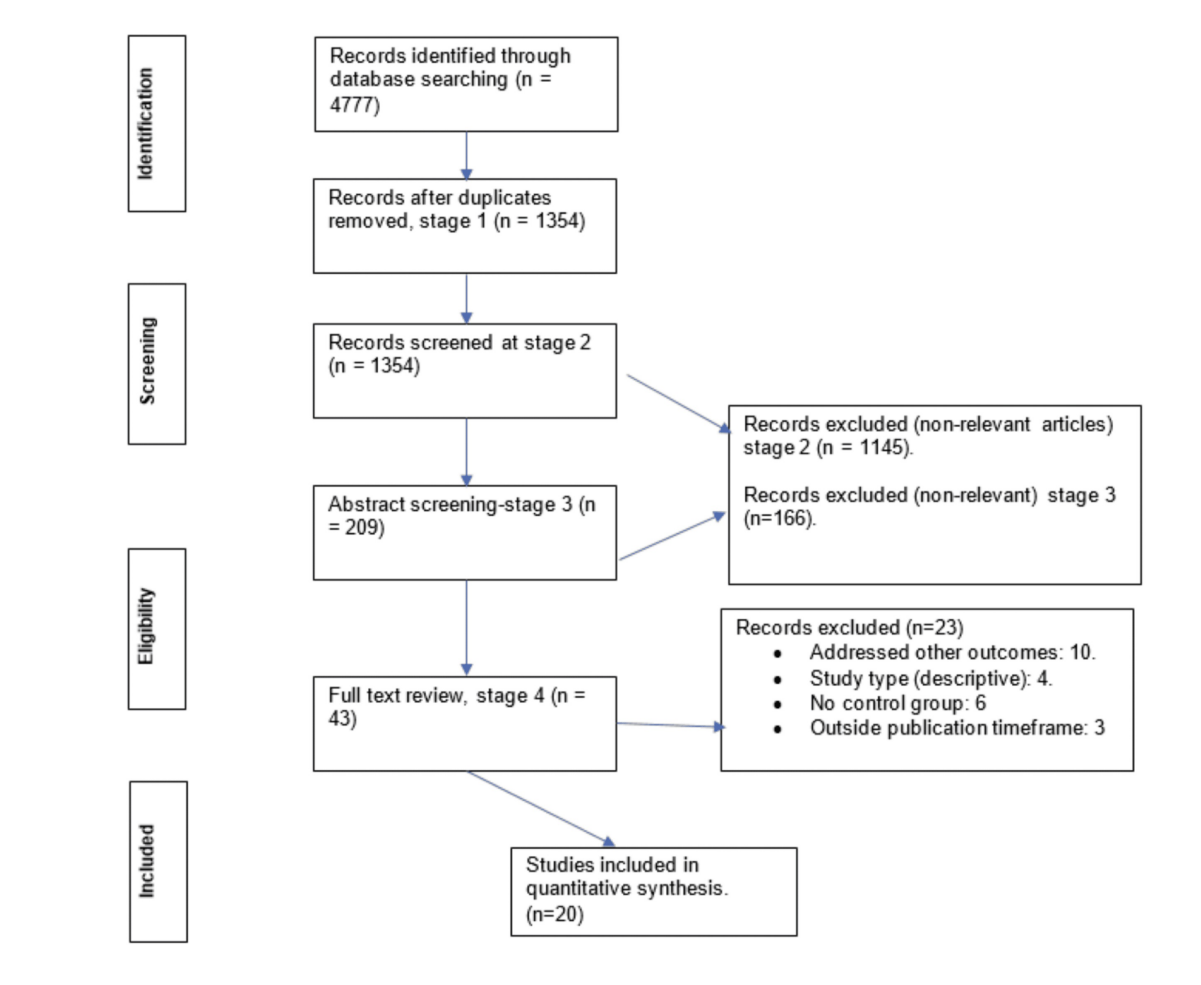
PRSIMA flow chart for study selection

A total of 4777 studies were retrieved after the search; 1354 articles remained after the removal of duplicates. As shown in Figure 1, 1311 articles were found to be irrelevant, while 23 articles were excluded for not meeting the inclusion criteria. Twenty articles were finally included in the narrative synthesis.

Glycemic control indices

Pegvisomant

The summary of studies assessing glycemic indices after medical therapy in acromegaly is shown in Table 1. In a randomized control trial by Trainer et al. [11], 56 partially responsive acromegaly patients were randomized to receive pegvisomant monotherapy or somatostatin analogues plus pegvisomant. Patients treated with somatostatin analogues only were used as controls. After a 40-week follow-up period, a significant decrease in glycemic parameters was observed in the pegvisomant monotherapy group. The fasting plasma glucose (FPG) decreased by -0.8, 95% CI (-1.16 to -0.53), while the 120-minute OGTT decreased by -2.9, 95% CI (-4.42 to -1.40). The HbA1c decreased by -0.40% 95% CI (-0.62% to -0.18%). In the somatostatin analogue plus pegvisomant group, no significant changes were observed in all three glycemic parameters. The SSA-only control group reported a significant rise in HbA1c of 0.14% 95% CI (0.005% to 0.23%), but the change in fasting plasma glucose and glucose tolerance was non-significant. The unblinded nature of the study resulted in a lower JBI score of 10.

**Table 1:** Studies on glycemic control

Author/year	Study design/setting	Participants N (median follow-up)	Medical therapies assessed	Mean Age	Outcomes measurement	Proportion with Diabetes	JBI Score
Barkan et al., 2005 [17]	Prospective open label (multicenter)	N=52 (32wk f/up)	Pegvisomant	49	FPG, HbA1c	25%	8/9
Berg C et al., 2010[18]	Prospective cohort, (Germany);	N=62 cases +655 controls (12 months f/up)	Pegvisomant	59	FPG HbA1c	37.6% cases; vs. 6.9% controls	8/11
Briet C et al., 2018 [19]	Retrospective cohort study (France);	N=96 (=/>62 months f/up)	Somatostatin analogs) vs. Pegvisomant	49;44;37	FPG	4%;4.3%;13.6%	9/10
Brue T et al., 2018 [20]	Multicenter retrospective analysis of ACROSTUDY data	N=1762 (4-year follow up)	Pegvisomant	Diabetes 46.7 Non-diabetes 39.7	FPG HbA1c	-	8/11
Caron P et al., 2016 [27]	PRIMARYS-open label multicenter (Europe).	N=90 (48-week f/up)	Lanreotide-Auto gel 120mg	54.7;47.6	FPG HbA1c	26.7% DM, (62.5% on drugs)	7/9
Chiloiro S et al., 2021 [21]	Retrospective cohort (multicenter);	N=40 (6 months f/up)	Pasireotide-LAR vs. Pasireotide-LAR+PEG	35.4	HbA1c	32.5% IGT, 22.5% DM/drugs	10/11
Colao A et al., 2009 [25,26]	Prospective cohort study (Italy).	N=100 (F/up 60 months),	SSAs alone, before or after surgery or surgery alone	51	FPG HbA1c GT	28% IGT 22% DM	9/11
Colao A et al., 2009 [25,26]	Prospective study	N=45 (5-year follow up)	Octreotide vs. Lanreotide		FPG	19% IFG 28% IGT 44% Diabetes	9/11
Colao A et al., 2014 [22]	RCT double blind (multicenter)	N=358 (12 months f/up)	Pasireotide vs. octreotide		HbA1c	44% pasireotide; 26.1% octreotide	12/13
Delaroudis S et al., 2008 [28]	Prospective Cohort study (Greece)	N=33 (6 months f/up)	Octreotide -LAR		FPG HbA1c	Not stated	7/10
Helseth S et al., 2016 [24]	RCT (post-hoc analysis of POTA trial-Norway)	N=55	Octreotide	47;45	FPG HbA1c GT	Not stated	7/13
Petersenn S et al., 2010 (extension 2014) [23]	RCT multicenter cross-over –phase II trial	N=60 (16-week f/up)	Octreotide100 mcg, vs. Pasireotide 200, 400, and 600 mcg	44.2	HbA1c	28% IFG 6.7% DM	9/13
Ronchi C et al., (2006) [30]	Retrospective cohort study (Italy)	N=69	Octreotide Lanreotide	44.1 cases 40.4 controls	HbA1c	-	10/11
Schmid H et al., 2016 [20]	RCT (exploratory analysis of PAOLA 3)	N=198 (24 weeks F/up)	Pasi-40mg vs. Pasi-60mg vs. octreotide LAR/Lanreotide autogel	-	HbA1c FPG	Pasi40-31% Pasi60-26%	10/13
Trainer P et al., 2009 [11]	RCT open label Multicenter	N=84 (40-week f/up)	PEG monotherapy vs. PEG+octreotide vs. octreotide-LAR	49;40;45	FPG GT HbA1c	40%;15%;15%	10/13
Tzanella M et al., 2011 [29]	Retrospective cohort, (Greece)	N=53(1 year follow-up)	Somatostatin analogs (SSA) vs. Surgery (TSS)	53. 48;4	FPG	40%	7/11

Another study by Barkan A et al. [17] investigated the safety parameters among patients with diabetes and those without diabetes when switched from octreotide-LAR to pegvisomant. This study had a follow-up period of 32 weeks. Diabetes patients reported a significant reduction in FPG from 7.4 to 5.0mmol/l (p < 0.05), and non-diabetes patients reported a significant reduction from 5.6 to 4.6mmol/l (p<0.001). HbA1c reduced from 7.8% to 6.3% (p<0.001) in diabetes patients and from 5.7% to 5.4% (p<0.0001) in non-diabetes patients. The study had a bias score of 8 owing to a lack of similarity between the two groups and uncontrolled confounders, including antidiabetic therapies.

A retrospective German study by Berg C et al. [18] investigated cardiovascular risk factors in acromegaly patients who had achieved IGF-1 normalization with pegvisomant as compared to healthy controls from the general population. After 12 months of PEG therapy, the study reported a significant HbA1c decline from (6.3+-1.0) % to (6.0+-1.0) % (p=0.02) in the pegvisomant-treated patients. The change in plasma glucose was not statistically significant. This study also scored eight on the JBI indicators due to dissimilar comparator groups and uncontrolled confounders in the two groups.

Briet C et al. [19] in a retrospective study in France compared glycemic outcomes in acromegaly patients who had achieved IGF-1 normalization with SSAs, pegvisomant, and pituitary surgery. The follow-up period for this study was approximately 70 months for all the study groups. The authors reported an overall improvement in FPG from 5.4 to 5.2 (p=0.026) in the entire cohort. However, patients achieving control by SSAs had a statistically significant increase in the FPG, while those on pegvisomant did not report any significant change. According to this study, surgically cured patients had a statistically significant decrease in FPG. It is noted, however, that there was a proportionately higher number of diabetes patients (13.6%) in the pegvisomant group compared to the surgical group (4%).

In a retrospective analysis of data from ACROSTUDY, Brue T et al. [20] assessed glycemic parameters in acromegaly patients with diabetes (DM) and those without diabetes (no-DM) who had been treated with pegvisomant. After a four-year follow-up period, the study reported a mean decrease in plasma glucose of 20.2 mg/ml in the DM group and a decrease of 3.9 mg/ml in the no-DM group (p<0.001). There was no significant change in the HbA1c in either group. The analysis also reported no significant correlation between IGF-1 levels and glycemic parameters. This retrospective study had a JBI risk of bias score of eight owing to the significant uncontrolled confounding between the two study groups.

Somatostatin Receptor Ligands

In a study by Chiloiro C et al. [21], the multicenter retrospective study compared pasireotide-LAR used alone or in combination with pegvisomant in 40 patients for a six-month period. This study reported a mean increase in HbA1c from baseline of 0.4 in the pasireotide 40 mg group and a mean HbA1c increase of 1.2 in the pasireotide 60 mg group (p=0.003). Combination therapy of pasireotide-LAR plus pegvisomant resulted in a non-significant HbA1c change of -0.3 from baseline.

In an exploratory analysis of the PAOLA trial (a comparison of pasireotide-LAR 40 mg and pasireotide-LAR 60 mg versus octreotide/lanreotide) (Schmid A et al.) [20], assessed glucose and GH levels after a 24-week follow-up period. This study reported a median increase in FPG of up to 14.9 mg/dl in the pasireotide-LAR 40 mg group and an increase of up to 30.7 mg/dl in the pasireotide-LAR 60 mg group. Median increase in the HbA1c in both pasireotide arms ranged between 11.1% in the low-dose and 21% in the high-dose groups, respectively. Blood glucose parameters in the control arm remained stable during the study.

Colao A et al. [22], in a randomized double-blind control trial, carried out a head-to-head superiority study of pasireotide versus octreotide. This study reported superior efficacy of pasireotide over octreotide after a 12-month follow-up period. Regarding glycemic parameters, the mean increase in HbA1c (SD) for pasireotide was reported as 0.87 (1.32) for diabetes patients, 0.64 (0.78) for those with pre-diabetes, and 0.75 (0.58) for non-diabetic participants. In the octreotide group, the mean increase in HbA1c (SD) was 0.03 (0.78), 0.11 (0.35), and 0.37 (0.46) in the three participant subgroups, respectively.

In an open-label crossover study of 60 patients, Petersen S et al. [23] assessed the efficacy and safety of pasireotide-LAR over a three-month period. Baseline FPG+/-SD was reported as 5.4+/-1.1 mmol/l and 6.4+/-2.3 mmol/l at the end of the study. Change in HbA1c was reported as 6.01+/-0.57% at baseline to 6.45+-0.84% at the end of the study period (p<0.001). This study had a JBI/RoB score of 9 out of 13 owing to a lack of randomization and blinding as well as a short follow-up period.

Helseth R et al. [24] assessed glucose parameters in acromegaly patients who received preoperative octreotide therapy as compared to those who had direct surgery in a post hoc analysis of the POTA (preoperative octreotide therapy for acromegaly) trial data. Baseline glucose indices were similar in both study groups. After three months of octreotide therapy, mean FPG and HbA1c remained the same at 5.7+/-0.9 mmol/l and 5.9+/-0.6%, respectively. For patients undergoing direct surgery, the mean FPG decreased from 5.6+/-0.8 mmol/l to 5.1+/-0.8 mmol/l. The HbA1c in the direct surgery group was 5.7+/-0.4% at baseline and 5.5+/0.4% at three months post-surgery. A subset of patients who did not achieve disease control after three months of octreotide therapy reported an increase in both FPG and HbA1c.

Colao A et al. [25,26] assessed cardiovascular parameters in 45 patients who received somatostatin analogues octreotide and lanreotide as initial therapy for a period of five years. In this study, change in FPG was reported as 5.84+/-1.43 mmol/l baseline to 5.20+/-0.39 mmol/l at the end of the study (p=0.11) for octreotide and 5.76+/-1.69 mmol/l baseline to 5.46+/-0.30 mmol/l at the end of the study (p=0.13) for lanreotide. Almost similar findings were reported in another study by Colao A et al. in the same year involving 100 patients with an equivalent follow-up duration of five years. In this second study, glucose parameters were assessed in four subgroups of patients. Group A received SRL therapy only, group B received SRL followed by surgery, group C received surgery only, and group D had surgery that was followed with SRL therapy. FPG decreased in group A from 5.78+/-1.63 mmol/l at baseline to 5.21+/-0.35 mmol/l at the end of the study (p=0.021). HbA1c was also significantly reduced in group A patients (p=0.013). In group B patients who received pre-surgical SSA therapy, FPG tended to increase from 5.22+/-0.76 mmol/l at baseline to 5.37+/-0.43 mmol/l at the end of the study (p=0.085). In a subgroup analysis of patients without diabetes, group B patients achieved a significant FPG rise from 5.05+/-0.46 mmol/l at baseline to 5.24+/-0.45 mmol/l at the end of the study (p=0.0039). Changes in FPG and HbA1c in all other groups were non-significant. Both studies by Colao A et al. [25,26], however, reported statistically significant decreases in both HOMA-R and HOMA-B indices.

The PRYMARYS trial was a 42-week single-arm open-label trial of Lanreotide auto gel 120 mg. In a post hoc analysis of the PRYMARYS data (Caron P et al.) [27] analyzed glucose and lipid parameters based on diabetes and disease control status. This analysis reported a significant HbA1c decrease of -1.44% (95% CI -2.56, -0.36) in the diabetes group only. No correlation was found between IGF-1 levels and glycemic indices.

Delaroudis S et al. [28] also assessed metabolic parameters after somatostatin analogue therapy but in patients with suboptimal control. This six-month prospective cohort study in Greece assessed 18 acromegaly patients treated with octreotide-LAR against 15 age-, sex-, and BMI-matched controls. The study reported a significant reduction in mean FPG from 6.75+/-0.55mmol/l at baseline to 5.92+/-0.47mmol/l at the end of the study (p=0.003). The mean HbA1c changed from 6.96+/-0.21% at baseline to 6.72+/-0.21% at the end of the study (p=0.001).

Tzanela et al. [29] also retrospectively assessed fasting plasma glucose in 30 acromegaly patients successfully controlled by somatostatin analogues (octreotide and lanreotide) compared to 20 patients controlled by surgery. In this study, the non-significant baseline and post-treatment FPG with somatostatin analogues were 6.45+/-0.5 mmol/l and 6.45+/-0.3 mmol, respectively. In the surgically controlled group, baseline FPG was 6.3+/-0.3 mmol/l and 4.7+/-0.2 mmol/l after treatment (p=0.0126).

Ronchi C et al. [30] retrospectively assessed long-term somatostatin (SSA) therapy with octreotide-LAR and lanreotide-autogel on disease control and cardiovascular parameters for over five years. This study demonstrated a rise in HbA1c from 4.7+/-0.7% to 5.7+/-0.5% (p<0.05) in patients with SSA-controlled disease, as well as a rise from 4.8+/-0.7% to 6.1+/-1.5% (p<0.05) for SSA-non-controlled disease.

Effect of acromegaly treatments on lipid profile

Pegvisomant

The studies assessing the impact of acromegaly treatments on lipid profile are summarized in Table 2. In this review, two studies assessed the impact of pegvisomant on lipid profiles. In the German study by Berg C et al. (2010), lipid parameters were assessed after IGF-1 normalization with pegvisomant over a 12-month period. The baseline lipid parameters were significantly higher (p<0.0001) in controls compared to cases with odds ratios (OR) for total cholesterol reported as 0.838 (95% CI, 0.800-0.877), LDLc OR 0.831 (95% CI, 0.787-0.877), and HDLc, 0.79 (95% CI, 0.35-0.850). The prevalence of dyslipidemia was also higher among the controls at 80% compared to 57% for the cases, (p<0.001) among the cases. Statin use at baseline was, however, higher among cases at 48% and 14% for control. After 12 months of treatment with pegvisomant, no significant change was observed in the lipid profiles among the acromegaly cases regardless of disease control status.

**Table 2:** Studies assessing lipid profile in acromegaly

Author/year	Design/setting	Participants/follow-up	M/F	Age	Intervention	Proportion of lipid therapy	Lipid profile outcomes	JBI score
Berg C. et al 2010 ^[18]^	Retrospective cohort (Germany)	N= 62 cases, 655 controls (12 months F/up)	31/31	58.8+-13.4	Pegvisomant	48% of cases; 14% of controls	No change	8/11
Briet, C. et al 2018 ^[19]^	Retrospective cohort (France)	N=96; surgery 51, SSA 23, PEG 22 (mean f/up 77 months)	39/47	Median 56	SSA PEG	Not stated	Significant changes observed in both groups	9/10
Caron, P. et al 2017 ^[27]^	Single arm open label (review of PRIMARYS data)	N=90 (48 weeks f/up) Controlled=30 non-controlled=58	43/47	49.5+-12.4	Lanreotide auto gel	Not stated	Significant change	7/9
Colao, A. et al 2009 ^[25,26]^	Retrospective cohort study (Italy)	n=45. octreotide n=28. Lanreotide n=17 (5-year f/up)	18/27	20-82	SSAs	Not stated	Significant change in both groups	9/11
Delaroudis, S. et al 2008 ^[28]^	Prospective cohort (Greece)	N=33. 18 cases (partially controlled disease), 15 healthy controls	8/10	48+-3.42	Octreotide-LAR	Not stated	Significant change in both groups	7/10
Ronchi, C. et al 2006 ^[30]^	Retrospective cohort; (Italy)	N=69. 36 SSA, Vs 33 cured surgically. (12 months f/up)	25/44	44.1+-14.3 cases. 40.4+-11.3 controls	SSA. Octreotide LAR- 16 Lanreotide- 20	66% dyslipidemia	Significant change in controlled disease	10/11

In the study by Briet C et al. [19], 22 patients in the pegvisomant arm who achieved IGF-1 normalization reported a significant rise in the LDLc from 2.87 (2.6-3.5) to 3.6 (2.9-4.3) (p=0.0007). Other lipid parameters did not change significantly, but there was a tendency towards a rise in total cholesterol.

Somatostatin Receptor Ligands

The study by Delaroudis S et al. [28] assessed lipid profiles in 18 partially controlled acromegaly patients after six months of octreotide-LAR therapy, compared to age- and sex-matched healthy controls. One-third of the patients were on statin therapy. This study reported a statistically significant fall in total cholesterol (mmol/l) from 6.33+/-0.4 to 5.85+/-0.3 (p=0.001), triglycerides (mmol/l) from 2.02+/-0.17 to 1.62+/-0.13 (p<0.001), and LDLc (mmol/l) from 4.42+/-0.36 to 4.06+/-0.31 (p=0.006), as well as a rise in HDLc (mmol/l) from 0.98+/-0.05 to 1.05+/-0.05 (p=0.017).

In the retrospective study in France by Briet C, et al. [19], patients who had achieved IGF-1 normalization after somatostatin analogue therapy also reported a significant rise in HDLc and a non-significant decrease in LDLc.

In the retrospective cohort study by Colao A et al. [25,26] assessing cardiac and metabolic parameters in acromegaly patients initially treated with octreotide (n=28) and lanreotide (n=17) for a five-year follow-up period, significant improvements in lipid parameters were reported in both groups. In the octreotide group, post-treatment lipid change for total cholesterol (mmol/l) was 5.17 to 4.81 (p=0.005), HDLc (mmol/l) from 1.20 to 1.40 (p=0.009), and triglycerides (mmol/l) from 1.68 to 1.17 (p=0.022). In the Lanreotide group, HDLc (mmol/l) increased from 1.11 to 1.40 (p<0.001), and triglycerides (mmol/l) decreased from 1.61 to 1.18 (p<0.001).

The post-hoc analysis of PRYMARYS data by Caron P et al. [27] assessed lipid parameters after 48 weeks of lanreotide autogel treatment. This analysis reported a marginally significant decrease in TGs and an increase in HDLc overall, regardless of diabetes or disease control status. Rates of lipid-lowering medications were, however, not reported in this study.

A retrospective study (n=69) by Ronchi C et al. [30] compared long-term (66 months) effects on disease control and cardiovascular parameters in acromegaly patients on somatostatin therapy against those controlled by surgery. Both study groups had similar baseline characteristics, including rates of dyslipidemia. For patients achieving disease control by somatostatin analogue therapy, a significant rise was reported in the HDLc (mg/dl) from 46+/-21 to 60+/-17 (p<0.05) and a fall in the LDLc level (mg/dl) from 144+/-49 to 125+/-37 (p<0.005). Changes in the total cholesterol and triglycerides in this group were not statistically significant. For patients not achieving disease control, no significant changes were observed in the lipid profile.

Blood pressure control

Pegvisomant

Summary data of studies that assessed the impact of medical treatments on blood pressure is shown in Table 3. In the German study by Berg C et al. [18], patients achieving disease control with pegvisomant reported a significant reduction in SBP from 141+/-27 mmHg to 135+/-17 mmHg (p=0.03). The change in diastolic blood pressure in patients with controlled disease was not statistically significant. When compared with patients with partially controlled disease, both SBP and DBP were significantly lower in controlled disease (SBP 141+/-4 mmHg versus 132+/-14 mmHg, p=0.04, and DBP 89+/-3 mmHg versus 84+/-2 mmHg, p=0.002). The prevalence of hypertension in this study was reported as 61% for acromegaly cases and 21% for the controls.

**Table 3:** Studies of the impact of acromegaly treatments on blood pressure

Author/year	Design/settings	Participants/follow up	M/F	Mean/median Age	Intervention	HTN/ drugs	Outcomes	ROB/JBI
Berg C et al. 2010 ^[18]^	Retrospective cohort study (Germany)	Cases n=62, controls n=655 (12 months f/up)	31/31	59+/-8	Pegvisomant	61% of cases; 21% of controls	Significant SBP decrease from baseline No change in DBP	8/11
Briet C et al. 2018 ^[19]^	Retrospective cohort study (France)	Surgery n= 51 (77 months f/up) SSA n=23 (75 months f/up) PEG n=22 (62 months f/up)	26/25; 12/11; 11/11	49 44 37	SSA PEG	Surgery; 23.5% SSA; 13% PEG; 13.6%	Significant decrease in SBP in Surgical group No change in the PEG group	9/10
Colao A et al. 2009 ^[25,26]^	Prospective cohort study (Italy)	Non-surgical acromegaly n=45. (5-year f/up)	18/27	Range 22-82	Octreotide Lanreotide	46.7%	Significant decrease in SBP and DBP in both groups.	9/11
Delaroudis S et al. 2008 ^[28]^	Prospective cohort study (Greece)	Partially controlled disease, n= 18. 15 controls (6 months f/up)	8/10	48	Octreotide-LAR	Not stated	Significant decrease in both SBP and DBP.	7/10
Ronchi et al. 2006 ^[30]^	Retrospective cohort (Italy)	n=69, SSA-Vs Surgically cured. (66 months f/up)	-	44; 40	Octreotide-LAR Lanreotide SR+ auto gel	55.6% of cases. 45% controls	No change in SSA group, Significant SBP decrease in surgically cured group.	10/11
Sardella C et al. 2014 ^[31]^	Retrospective cohort study (Italy)	n=58, (24 months f/up)	19/39	49.4	Lanreotide Octreotide SSA+PEG PEG	52% HTN-80% of HTN on drugs	Significant increase in SBP and DBP in uncontrolled normotensive acromegaly. No change in controlled and hypertensive acromegaly.	9/11

In another retrospective study, Sardella et al. (2014) [31] investigated the impact of different acromegaly treatments in normotensive and hypertensive patients (n=58). 52% of the study group were hypertensive, with 80% taking hypertension medications. Acromegaly treatments comprised Lanreotide (n=18) or Octreotide (n=13), SRL plus pegvisomant (n=19), and pegvisomant monotherapy (n=8). After 24 months of follow-up, normotensive patients with uncontrolled acromegaly reported a statistically significant rise in both mean SBP of 25 mmHg (15-30), p < 0.001, and mean DBP of 15 mmHg (15-30 mmHg), p < 0.001. In the hypertensive and controlled groups, no significant changes were observed.

In the study by Briet C et al. (2018) [19] assessing cardiovascular and metabolic parameters after achieving IGF-1 normalization, a significant mean decrease in SBP was observed overall and in patients controlled with surgery, but not in those controlled with pegvisomant.

Somatostatin Receptor Ligands (SRLs)

Ronchi C et al. [30] retrospectively assessed the long-term impact of octreotide LAR and lanreotide-SR (SRLs) on cardiovascular parameters in acromegaly. Both systolic and diastolic blood pressure were determined at baseline and after a median follow-up of 66 months. Prevalence of HTN was reported as 55.6% for the cases and 45% for the controls. No significant change was observed for both systolic and diastolic blood pressure with SRL therapy for both controlled and uncontrolled disease.

The study by Colao A et al. [25,26], also retrospectively assessed changes in systolic and diastolic blood pressure in 45 acromegaly patients receiving octreotide-LAR or lanreotide-LAR after a five-year follow-up period. In this study, the prevalence of HTN was reported as 46% overall. In the octreotide group, the mean SBP changed from 152+/-22 mmHg at baseline to 145+/-20 mmHg at study end (p=0.014), while the mean DBP changed from 93+/-11 mmHg at baseline to 85+/-6 mmHg at the end of the study (p=0.0014). In the Lanreotide group, the mean SBP changed from 156+/-23 mmHg baseline to 152+/-19 mmHg (p=.0053), while the mean DBP changed from a baseline of 96+/-11 mmHg to 88+/-7 mmHg (p<0.0001) at the end of the study period.

In the prospective cohort study conducted in Greece by Delaroudis S et al. [28] on the impact of partial disease control with octreotide-LAR, a significant decrease was observed in both the systolic and diastolic blood pressure after a six-month follow-up. The study reported a decrease in mean SBP from 139.17+/-4.99 mmHg at baseline to 131.67+/-5.39 mmHg (p=0.001) at the end of the study. The mean DBP changed from 85.00+/-2.39 mmHg at baseline to 79.72+/-2.57 mmHg (p=0.001) at the end of the follow-up period. The rates of hypertension and drug usage were, however, not reported by this study.

Left Ventricular Mass After Acromegaly Treatment

Four studies in this review evaluated the change in LVMI after acromegaly medical treatments (Table 4). Andreassen et al. [32] conducted a prospective cohort study with eight cases and eight healthy age- and sex-matched controls over a three-month period. The patients were treated with octreotide-LAR, pegvisomant mono, and PEG+ octreotide. The investigators assessed LV mass using cardiac magnetic resonance imaging (CMRI). At baseline, the mean left ventricular mass index (LVMi) for cases was 92 (53-161) g/m² and that for controls was 62 (49-76) g/m² (p=0.016). After three months of treatment, LVMi for cases changed to 87 (58-175) g/m² (p=0.54). This study had an overall JBI risk of bias score of 7/11 owing to the lack of similarity between cases and controls, poor control of confounding factors, and a very short follow-up period.

**Table 4:** Impact of medical therapies on LV mass

Author/year	Design/setting	Participant (Follow up)	Mean age(years)	Medical therapies assessed	Cardiac study	Outcomes	Quality of evidence score
Andreassen M et al. 2010 ^[32]^	Cohort prospective (Denmark)	N=16 (3 months F/up)	53+-12	Octreotide LAR Pegvisomant monotherapy Pegvisomant + octreotide LAR	CMRI	No change in LVMi in all groups	JBI =7/11
De-Alcubierre et al. 2024 ^[33]^	Case-control (Italy)	N=37	50	SSAs SSA+PEG Pasireotide	CMRI	LVMi in cured acromegaly same as control group	JBI=10/10
De-Marinis et al. 2008 ^[34]^	Prospective cohort study (Italy)	N= 48 (12 months f/up)	Cases;54.6. controls 46.2	Octreotide + surgery. Surgery alone	Echo	Significant decrease in LVMi in both groups	JBI=10/11
Colao A et al. 2009 ^[25,26]^	Retrospective cohort (Italy)	N=45 (5-year f/up)	22-82	Octreotide Lanreotide	Echo	Significant decrease in LVMi in both groups	JBI=9/11

De Alcubierre et al. [33] carried out a case-control study comparing active cases on treatment with SSAs (octreotide and lanreotide), SSA+PEG, or pasireotide with patients with non-functioning adrenal adenomas as controls. The mean LVMi for patients with active disease was 62+/-12.1 g/m² and that for patients with cured disease was 45.5+/-12.5 g/m² (p=0.009). The study also demonstrated a higher LVMi among active cases compared to the control group, which had a mean LVMi of 43.5+/-8.5 g/m² (p=0.001). There was also reported a positive correlation between LVMi and IGF-1 levels (r=0.600, p=0.009). The study had a high JBI score of 10/10 for its category.

Using Doppler echocardiography, the study by De Marinis, L., et al. [34] sought to determine cardiovascular features in patients with medically controlled acromegaly with octreotide against those cured by surgery. After a follow-up period of 12 months, the LVMi among surgically cured patients changed from 152.5+/-9.1 g/m² at baseline to 116+/-9.5 g/m² at the study end (p<0.05). For patients achieving control after surgery plus somatostatin analogue therapy, the LVMi changed from 140.8+/-5.7 g/m² at baseline to 110.9+/-6.1 g/m² at the end of the study (p<0.05). This prospective cohort study had a low risk of bias with a JBI score of 10/11.

In the five-year retrospective study by Colao A et al. [25,26], cardiac parameters were assessed using Doppler echocardiography in patients treated with octreotide-LAR or lanreotide-LAR. In the octreotide-treated patients, LVMI decreased from 157.6 +/- 64 g/m² at baseline to 123.2 +/- 38 g/m² at study end (p=0.0046). In the Lanreotide group, the LVMi decreased from 187.0+/-33 g/m² at baseline to 144.2+/-42.2 g/m² at the end of the follow-up period (p<0.0001).

Discussion

Acromegaly patients have a significant burden of diabetes mellitus, and control of diabetes is one of the key objectives of treatment. The pathophysiology of diabetes in acromegaly is thought to be the result of disease-related factors causing an increase in insulin resistance from increased free fatty acids. Additionally, suppression of beta-cell function from glucolipotoxicity and attendant somatostatin receptor ligand treatments exacerbates the glycemic status (Moustaki M et al.) [35].

In this review, most studies have shown a beneficial effect of pegvisomant on lowering FPG and HbA1c in both diabetic and non-diabetic patients. The beneficial effect of pegvisomant on glycemic indices is thought to result from the suppression of lipolysis with a consequent increase in insulin sensitivity, Lindberg-Larsen R et al. [36], Moustaki M et al. [35]. When used in combination with somatostatin receptor ligands, the beneficial effect of pegvisomant on the glycemic indices is blunted or ceases to appear. This effect, however, also raises the possibility of ameliorating the hyperglycemic effect of SRLs, especially pasireotide, with the addition of pegvisomant in patients with uncontrolled acromegaly.

A significant number of participants in this review had diabetes and most likely took medications to lower FPG and HbA1c. While this effect may have been controlled in the randomized studies, most of the prospective and retrospective studies did not report control measures for this potential confounding factor.

The multi-receptor SRL pasireotide was shown to be associated with an increase in FPG and HbA1c by all the included studies. This effect was shown to be enhanced at higher doses of pasireotide and among non-responding patients. From previous studies, the hyperglycemic effect of pasireotide has been attributed to the suppression of both beta-cell function and a decrease in incretin levels (Moustaki M et al.) [35].

Studies on first-generation SRLs octreotide and lanreotide have, however, shown inconsistent results on their glycemic impact regardless of duration of treatment, disease control, or diabetes status. Caron P et al. [27] reported improvement in glycemic indices only in diabetes patients, which could have been confounded by diabetes treatments, while two other long-term studies, Colao A et al. [25,26] and Ronchi C et al. [30], reported neutral and worsening glycemic parameters, respectively. Only one short-term study by Delaraudis et al. [28] reported improvement of glycemic indices with octreotide. The current findings are consistent with a meta-analysis by Mazziotti G et al. [37], which reported a significant reduction in fasting plasma insulin by first-generation SRLs but no significant impact on both FPG and HbA1c. This study concluded that the effect of this group of drugs on glycemic parameters in acromegaly is minor.

The effect of growth hormone (GH) in adipose tissue is to cause lipolysis with the resultant increase in free fatty acids and a decrease in visceral adipose tissue. This has been thought to be part of the survival mechanism to switch substrate consumption from glucose and protein to lipids during the fasting state, Moller N & Jorgensen J [38]. Parkinson C et al. [39] and Wang M et al. [40] reported that patients with acromegaly have lower baseline total cholesterol, HDLc, and LDLc levels and higher triglyceride levels compared to the healthy population, which has been attributed to the lipolytic effects of GH. These findings are consistent with those of the pegvisomant study by Berg C et al. [18].

Putatively, therefore, effective disease control would result in an increase in adiposity and lipid parameters, as was shown in the study by Reyes-Vidal et al. [15]. In this review, Berg C et al. [18] reported non-significant changes in lipid profiles in pegvisomant-treated patients, while Briet et al. [19] reported a rise in LDLc after pegvisomant. These findings are consistent with earlier findings by Parkinson C et al. [39], who reported a rise in TC and LDLc after IGF-1 normalization with pegvisomant.

Regarding SRL therapy and lipid profiles, there is a general trend for the improvement of various parameters of the lipid profile in the reviewed studies. This effect was observed in patients achieving complete disease control (Briet C et al.) [19] as well as those who only achieved partial control (Delaroudis S et al.) [28]. It can be hypothesized that the beneficial effect of SRLs on lipid profile may be the result of a direct effect of these agents on lipid metabolism. This is supported by in-vitro studies that have demonstrated a direct effect of SRLs on adipocytes (Zhao Z et al. [41]).

Statins have a significant impact on lipid parameters in acromegaly patients (Mishra M et al. [42]). A potential limitation for this review is therefore the confounding effects of lipid-lowering medications or measures. None of the studies assessing SRLs and lipid profiles in this review were randomized, and neither study reported any measures to control for this potential confounding factor.

The prevalence of LVH in acromegaly patients has been estimated at between 50 and 80% and has been shown to be determined by the patient's age, duration of disease, BMI, SBP, gender, and GH/IGF-1 levels (Vitale G et al.) [4]. The increase in the left ventricular mass in acromegaly patients is thought to be largely due to the direct effect of GH and IGF-1 on cardiomyocytes and to a small extent, the result of preexisting hypertension (Sharma M et al.) [43].

In a study by Toumanidis et al. [44], it was shown that patients with active acromegaly disease had a higher left ventricular mass index compared to those with inactive/controlled disease. Long-term studies in this review have shown a similar impact of medical therapies on LVMi in patients who achieved disease control or significant reduction in IGF-1 levels De Alcubierre et al. [33], De-Marinis et al. [34], and Colao A et al. [25,26]. An earlier meta-analysis by Maison P et al. [45] had also shown significant improvement in cardiac morphology and function after somatostatin analogue therapy with strict control of GH/IGF-1 levels.

Other single-arm open-label prospective studies (not included in this synthesis) also demonstrated a reduction in LVMi with medical therapies of lanreotide-autogel, pegvisomant, and a combination of SRL and pegvisomant (Annamalai A et al. [46], Pivonello et al. [47] and Auriemma R et al. [48]). According to Sharma M et al. [43], the effect of SRLs on LV mass may also be mediated directly through somatostatin receptors on the cardiac myocytes. Whether this impact is also influenced by gender, as suggested by Annamalai A et al. [46], or other variables such as blood pressure control or LV remodelling medications is a subject for future investigations.

One study in this review failed to show a significant change in the LVMi index after medical treatment (Andreasen M et al.) [32]. This may be explained by a lack of sufficient power due to the small sample size used, and the study period of three months may have also been too short for significant remodelling changes to be observed. It should also be noted that while this study reported a significant reduction in IGF-1 levels compared to baseline, the IGF-1 levels for cases remained much higher than the controls.

A combination of pathophysiologic mechanisms has been proposed for the development of hypertension in acromegaly patients. These include GH-induced volume expansion, overactivity of the renin-angiotensin-aldosterone system, endothelial dysfunction, and increased sympathetic tone (Sharma M et al.) [43]. As has been alluded to by the review by Wolters C et al. [49], the relationship between IGF-1 and cardiovascular disease is non-linear, and the deleterious effects of prolonged excess GH/IGF-1 on the endothelium may not be completely reversed even with disease control. An observational study found a positive correlation between blood pressure and IGF-1 only in patients with active disease (Fedrizi D et al.) [50].

In this review, one study reported a significant decrease in SBP with pegvisomant (Berg C et al.) [18], while two studies reported a significant decrease in both SBP and DBP with somatostatin receptor ligands (Colao A et al.) [25,26] and Delaroudis S et al. [28]. It is, however, noted that none of these studies were randomized, and there is a potential confounding effect of concomitant antihypertensive treatments. Further studies with randomization and blinding might therefore shed more light on the actual impact of acromegaly medical treatments on blood pressure.

One of the notable limitations of this review is that most of the included studies were predominantly from a few European centres. This may affect the overall generalizability of the findings to other populations. Another limitation is that the review is also relatively broad in scope and heterogeneity and therefore could not allow for a focused meta-analysis of the data. However, the review is strong because it has assessed the basic clinical parameters that are used by the majority of practitioners on a day-to-day basis. This gives the review direct clinical relevance and applicability. The review also provides a strong foundation for future targeted hypothesis testing and meta-analysis in some of these clinical parameters.

## Conclusions

This review has established convincing evidence for improvement of glycemic parameters with pegvisomant therapy and worsening of the same for patients treated with pasireotide. There is also good evidence for long-term benefits for both pegvisomant and somatostatin receptor ligands for the reduction of left ventricular mass, especially after the achievement of disease control. Somatostatin receptor ligands seem to have a better lipid-lowering effect compared to pegvisomant. However, the effect of the current medical therapies on blood pressure remains unclear. This review therefore reinforces the recommendation for individualized therapy based on the patient's comorbidity and cardiovascular risk profile.

## References

[1] Chanson P, Salenave S (2008). Acromegaly. Orphanet J Rare Dis.

[2] Ogedegbe OJ, Cheema AY, Khan MA (2022). A comprehensive review of four clinical practice guidelines of acromegaly. Cureus.

[3] Puglisi S, Ferraù F, Ragonese M (2020). Cardiometabolic risk in acromegaly: a review with a focus on pasireotide. Front Endocrinol (Lausanne).

[4] Vitale G, Galderisi M, Pivonello R Prevalence and determinants of left ventricular hypertrophy in acromegaly: impact of different methods of indexing left ventricular mass.

[5] Ershadinia N, Tritos NA (2022). Diagnosis and treatment of acromegaly: an update. Mayo Clin Proc.

[6] Sandret L, Maison P, Chanson P (2011). Place of cabergoline in acromegaly: a meta-analysis. J Clin Endocrinol Metab.

[7] Carmichael JD, Bonert VS, Nuño M (2014). Acromegaly clinical trial methodology impact on reported biochemical efficacy rates of somatostatin receptor ligand treatments: a meta-analysis. J Clin Endocrinol Metab.

[8] Gadelha MR, Kasuki L, Korbonits M (2013). Novel pathway for somatostatin analogs in patients with acromegaly. Trends Endocrinol Metab.

[9] Caron PJ, Bevan JS, Petersenn S (2014). Tumor shrinkage with lanreotide Autogel 120 mg as primary therapy in acromegaly: results of a prospective multicenter clinical trial. J Clin Endocrinol Metab.

[10] Bronstein MD, Fleseriu M, Neggers S (2016). Switching patients with acromegaly from octreotide to pasireotide improves biochemical control: crossover extension to a randomized, double-blind, Phase III study. BMC Endocr Disord.

[11] Trainer PJ, Ezzat S, D'Souza GA (2009). A randomized, controlled, multicentre trial comparing pegvisomant alone with combination therapy of pegvisomant and long-acting octreotide in patients with acromegaly. Clin Endocrinol (Oxf).

[12] van der Lely AJ, Biller BM, Brue T (2012). Long-term safety of pegvisomant in patients with acromegaly: comprehensive review of 1288 subjects in ACROSTUDY. J Clin Endocrinol Metab.

[13] Giustina A, Barkhoudarian G, Beckers A (2020). Multidisciplinary management of acromegaly: A consensus. Rev Endocr Metab Disord.

[14] Holdaway IM, Rajasoorya RC, Gamble GD Factors influencing mortality in acromegaly.

[15] Reyes-Vidal C, Fernandez JC, Bruce JN (2014). Prospective study of surgical treatment of acromegaly: effects on ghrelin, weight, adiposity, and markers of CV risk. J Clin Endocrinol Metab.

[16] Maola S, Munn Z, Tufanaru C (2020). Systematic reviews of etiology and risk. JBI Man Evi Syn.

[17] Barkan AL, Burman P, Clemmons DR (2005). Glucose homeostasis and safety in patients with acromegaly converted from long-acting octreotide to pegvisomant. J Clin Endocrinol Metab.

[18] Berg C, Petersenn S, Lahner H (2010). Cardiovascular risk factors in patients with uncontrolled and long-term acromegaly: comparison with matched data from the general population and the effect of disease control. J Clin Endocrinol Metab.

[19] Briet C, Ilie MD, Kuhn E Changes in metabolic parameters and cardiovascular risk factors after therapeutic control of acromegaly vary with the treatment modality. Data from the Bicêtre cohort, and review of the literature. Endocrine.

[20] Schmid HA, Brue T, Colao A (2016). Effect of pasireotide on glucose- and growth hormone-related biomarkers in patients with inadequately controlled acromegaly. Endocrine.

[21] Chiloiro S, Giampietro A, Visconti F (2021). Glucose metabolism outcomes in acromegaly patients on treatment with pasireotide-LAR or pasireotide-LAR plus Pegvisomant. Endocrine.

[22] Colao A, Bronstein MD, Freda P (2014). Pasireotide versus octreotide in acromegaly: a head-to-head superiority study. J Clin Endocrinol Metab.

[23] Petersenn S, Schopohl J, Barkan A (2010). Pasireotide (SOM230) demonstrates efficacy and safety in patients with acromegaly: a randomized, multicenter, phase II trial. J Clin Endocrinol Metab.

[24] Helseth R, Carlsen SM, Bollerslev J (2016). Preoperative octreotide therapy and surgery in acromegaly: associations between glucose homeostasis and treatment response. Endocrine.

[25] Colao A, Auriemma RS, Galdiero M (2009). Impact of somatostatin analogs versus surgery on glucose metabolism in acromegaly: results of a 5-year observational, open, prospective study. J Clin Endocrinol Metab.

[26] Colao A, Auriemma RS, Galdiero M (2009). Effects of initial therapy for five years with somatostatin analogs for acromegaly on growth hormone and insulin-like growth factor-I levels, tumor shrinkage, and cardiovascular disease: a prospective study. J Clin Endocrinol Metab.

[27] Caron PJ, Petersenn S, Houchard A (2017). Glucose and lipid levels with lanreotide autogel 120 mg in treatment-naïve patients with acromegaly: data from the PRIMARYS study. Clin Endocrinol (Oxf).

[28] Delaroudis SP, Efstathiadou ZA, Koukoulis GN (2008). Amelioration of cardiovascular risk factors with partial biochemical control of acromegaly. Clin Endocrinol (Oxf).

[29] Tzanela M, Vassiliadi DA, Gavalas N (2011). Glucose homeostasis in patients with acromegaly treated with surgery or somatostatin analogues. Clin Endocrinol (Oxf).

[30] Ronchi CL, Varca V, Beck-Peccoz P (2006). Comparison between six-year therapy with long-acting somatostatin analogs and successful surgery in acromegaly: effects on cardiovascular risk factors. J Clin Endocrinol Metab.

[31] Sardella C, Urbani C, Lombardi M (2014). The beneficial effect of acromegaly control on blood pressure values in normotensive patients. Clin Endocrinol (Oxf).

[32] Andreassen M, Faber J, Kjær A (2010). Cardiac effects of 3 months treatment of acromegaly evaluated by magnetic resonance imaging and B-type natriuretic peptides. Pituitary.

[33] De Alcubierre D, Feola T, Cozzolino A (2024). The spectrum of cardiac abnormalities in patients with acromegaly: results from a case-control cardiac magnetic resonance study. Pituitary.

[34] De Marinis L, Bianchi A, Mazziotti G (2008). The long-term cardiovascular outcome of different GH-lowering treatments in acromegaly. Pituitary.

[35] Moustaki M, Paschou SA, Xekouki P (2023). Secondary diabetes mellitus in acromegaly. Endocrine.

[36] Lindberg-Larsen R, Møller N (2007). The impact of pegvisomant treatment on substrate metabolism and insulin sensitivity in patients with acromegaly. J Clin Endocrinol Metab.

[37] Mazziotti G, Floriani I, Bonadonna S (2009). Effects of somatostatin analogs on glucose homeostasis: a metaanalysis of acromegaly studies. J Clin Endocrinol Metab.

[38] Møller N, Jørgensen JO (2009). Effects of growth hormone on glucose, lipid, and protein metabolism in human subjects. Endocr Rev.

[39] Parkinson C, Drake WM, Wieringa G (2002). Serum lipoprotein changes following IGF-I normalization using a growth hormone receptor antagonist in acromegaly. Clin Endocrinol (Oxf).

[40] Wang M, Guo S, He M (2020). High-performance liquid chromatography-mass spectrometry-based lipid metabolite profiling of acromegaly. J Clin Endocrinol Metab.

[41] Zhao Z, Gong F, Duan L (2022). Somatostatin receptor ligands suppressed proliferation and lipogenesis in 3T3-L1 preadipocytes. Basic Clin Pharmacol Toxicol.

[42] Mishra M, Durrington P, Mackness M (2005). The effect of atorvastatin on serum lipoproteins in acromegaly. Clin Endocrinol (Oxf).

[43] Sharma MD, Nguyen AV, Brown S, Robbins RJ (2017). Cardiovascular disease in acromegaly. Methodist Debakey Cardiovasc J.

[44] Toumanidis ST, Evangelopoulos ME, Ilias I (2011). Is left ventricular dysfunction reversed after treatment of active acromegaly?. Pituitary.

[45] Maison P, Tropeano AI, Macquin-Mavier I (2007). Impact of somatostatin analogs on the heart in acromegaly: a metaanalysis. J Clin Endocrinol Metab.

[46] Annamalai AK, Webb A, Kandasamy N (2013). A comprehensive study of clinical, biochemical, radiological, vascular, cardiac, and sleep parameters in an unselected cohort of patients with acromegaly undergoing presurgical somatostatin receptor ligand therapy. J Clin Endocrinol Metab.

[47] Pivonello R, Galderisi M, Auriemma RS (2007). Treatment with growth hormone receptor antagonist in acromegaly: effect on cardiac structure and performance. J Clin Endocrinol Metab.

[48] Auriemma RS, Grasso LF, Galdiero M (2017). Effects of long-term combined treatment with somatostatin analogues and pegvisomant on cardiac structure and performance in acromegaly. Endocrine.

[49] Wolters TL, Netea MG, Riksen NP (2020). Acromegaly, inflammation and cardiovascular disease: a review. Rev Endocr Metab Disord.

[50] Fedrizzi D, Rodrigues TC, Costenaro F (2011). Hypertension-related factors in patients with active and inactive acromegaly. Arq Bras Endocrinol Metabol.

